# Strategies for Preventing HIV Infection Among HIV-Uninfected Women Attempting Conception with HIV-Infected Men — United States

**DOI:** 10.15585/mmwr.mm6621a2

**Published:** 2017-06-02

**Authors:** Jennifer F. Kawwass, Dawn K. Smith, Dmitry M. Kissin, Lisa B. Haddad, Sheree L. Boulet, Saswati Sunderam, Denise J. Jamieson

**Affiliations:** ^1^Division of Reproductive Health, National Center for Chronic Disease Prevention and Health Promotion, CDC; ^2^Division of Reproductive Endocrinology and Infertility, Department of Gynecology and Obstetrics, Emory University School of Medicine, Atlanta, Georgia; ^3^Division of HIV/AIDS Prevention, National Center for HIV/AIDS, Viral Hepatitis, STD, and TB Prevention, CDC.

By the end of 2014, a total of 955,081 persons in the United States (299.5 per 100,000 population) had received a diagnosis of human immunodeficiency virus type 1 (HIV-1) infection (*1*). The annual estimated number of HIV infections and incidence rate in the United States decreased from 2010 to 2014, and the survival rate has increased over time (*1*). Effective highly active antiretroviral therapy (HAART) is helping persons with HIV to live longer, healthier lives. Many of these persons, including an unknown percentage in discordant relationships (i.e., one partner is HIV-infected, and the other is HIV-uninfected), might wish to have their own biologic children. When the female partner is HIV-infected and the male partner is not, a discordant couple can undergo autologous sperm intrauterine inseminations to achieve conception without placing the man at risk for infection. However, for HIV-discordant couples in which the man is HIV-infected and the woman is not, strategies to minimize the risk for sexual transmission are needed. In 1988, CDC recommended against insemination with semen from HIV-infected men (*2*). Since 1988, new information has emerged regarding prevention of HIV transmission in HIV-discordant couples. This report reviews laboratory and epidemiologic information regarding the prevention of HIV transmission for HIV-discordant couples, in which the male is HIV-infected and the female is HIV-uninfected, who would like to attempt conception.

Insemination with sperm from an HIV-negative donor is the safest option for an HIV-uninfected woman to conceive with an HIV-infected male partner. However, risk-reducing approaches using sperm from an HIV-infected male partner do exist. One strategy is the use of viral suppression with HAART for the male partner, with intercourse without condom protection limited to the time around ovulation, while the female partner is taking daily oral antiretroviral preexposure prophylaxis (PrEP) (*3*). Another strategy that can be used in conjunction with HAART and PrEP is collection and washing of the male partner’s sperm to remove cells infected with HIV, followed by testing to confirm the absence of HIV prior to intrauterine insemination (IUI) of the female partner or in vitro fertilization (IVF) (*4*). Each method has a unique risk profile, might confer distinctive advantages and disadvantages, and requires varying degrees of assistance from the medical community. Before attempting conception, discordant couples might wish to discuss treatment options with an experienced medical provider who can relay the risks and benefits of each treatment modality as it applies to the couple’s specific situation.

## Background

The American College of Obstetricians and Gynecologists, the American Society of Reproductive Medicine, and others have published guidance documents that emphasize the importance of considering HIV a chronic disease or disability, which should not result in discrimination and for which fertility treatment should be offered if it is desired (*5*,*6*). Access to treatments that require the assistance of a physician might be limited by financial and legal barriers. These barriers include state laws that preclude the use of HIV-positive sperm or fear of liability if seroconversion occurs, physician reluctance to treat discordant couples, and concerns based on previous publications, including those from CDC, that warned against use of sperm from HIV-infected men for insemination (*2*,*5*–*7*). Whereas HIV-infected men who are currently under the care of a physician are likely already receiving HAART, their sexual partners might or might not be using PrEP.

## Rationale and Evidence

For HIV-discordant couples (HIV-infected male and HIV-uninfected female) who want to conceive, considerations in choosing the optimal method to achieve pregnancy include transmission risk, treatment efficacy, and affordability. Use of HIV-negative donor sperm that meets Food and Drug Administration donor eligibility criteria remains the safest option for avoiding HIV infection of the female partner (*2*,*8*). Recent evidence suggests that discordant couples who wish to have their own biologic children might consider using condomless intercourse timed to coincide with ovulation, or IUI or IVF in combination with sperm washing (*4*). Avoidance of HIV transmission is optimized when the male partner is virologically suppressed on HAART and the female partner is on PrEP (*3*). Further considerations apply when the couple has infertility issues. Many men with HIV infection have altered semen parameters that make insemination or IVF the optimal form of fertility treatment (*9*). Moreover, female infertility factors such as tubal disease might warrant a particular treatment, for example, IVF. Thus, testing for potential causes of infertility (such as tubal factors, male factors, and ovulatory dysfunction) is a reasonable early step in treating all HIV-discordant couples who desire conception.

Condomless intercourse is associated with the highest risk for HIV transmission. The risk for male-to-female transmission in HIV-discordant couples has been estimated as approximately 1–2 per 1,000 episodes of condomless intercourse (*10*). This estimation of risk is based, however, on natural history studies of couples before routine availability of HIV viral load measurements and HAART, and might vary widely with characteristics of the man and woman, including the presence of other sexually transmitted diseases, inflammation within the genital tract, and viral load of the infected partner (*10*). Among men on HAART with undetectable seminal and plasma viral loads, the postulated risk for transmission to a female partner during condomless intercourse is low (0.16 per 10,000 exposures, 95% confidence intervals [CI] = 0.02–1.3) (*10*). However, although some studies suggest a parallel reduction in plasma and semen viral loads (*11*), other evidence suggests that plasma and semen viral loads might not correlate (*12*); men with undetectable plasma viral loads have had virus isolated from their semen (*13*). As a result, men on HAART with undetectable plasma viral loads might still be at some (albeit, very low) risk (1.2 per 100 person-years, CI = 0.9–1.7) for transmitting HIV-1 to their female partner through condomless sexual intercourse (*14*). In addition to viral suppression with HAART, the risk for sexual transmission can be further reduced by minimizing exposure frequency and limiting condomless intercourse to time of ovulation, thereby maximizing the chance of conception, and by use of PrEP by the uninfected partner (*3*).

Recent data exist on the safety of IUI following sperm washing (*4*). Sperm washing methods have evolved to include a two-step process including gradient centrifugation and separation of sperm from semen followed by use of a lymphocyte preparation medium that requires motile sperm to swim up and separate from lymphocytes, which are the largest reservoir of virus in semen (*15*). Whether HIV can infect spermatozoa is not clear; some studies suggest that HIV-1 can infect spermatozoa (*16*,*17*), whereas others refute these findings (*18*,*19*). Testing of the resultant washed specimens by polymerase chain reaction (PCR), real-time PCR (qPCR), and nucleic acid sequence based amplification for HIV RNA have suggested that 92%–99% of specimens of processed semen contain no virus measurable above the limits of detection of the test (*4*,*20*–*23*). Testing the resultant specimen for presence of residual HIV before insemination can identify 1.3%–7.7% of specimens that have been noted to be positive after appropriate washing (*4*,*20*–*24*). These 1.3%–7.7% of washed specimens that test positive even after washing would be discarded and not used for insemination.

Evidence suggests that these newer methods of sperm washing significantly reduce the risk for HIV-1 transmission (*25*,*26*). Approximately 11,500 assisted conception (IUI and IVF) cycles in women without HIV infection using sperm separated from semen of their HIV-infected partners have been reported with zero HIV transmissions to the women or resultant offspring (*4*,*22*,*23*,*27*–*29*). In couples using IUI with sperm washing, risk can presumably be further reduced with the use of HAART by the infected partner and the use of PrEP by the uninfected partner (*3*).

There are some reports of women becoming HIV-infected at some point after IUI; however, the evidence suggests that the infections resulted from subsequent condomless intercourse with their infected partner (*29*). In a 1990 report describing HIV infection in a woman who underwent IUI using processed semen from her HIV-infected husband, CDC recommended against the use of sperm from an HIV-infected partner; the mechanism of sperm preparation at the time was determined to not effectively separate lymphocytes from spermatozoa (*7*). Seroconversion was also reported 4 years after IUI in a woman who was HIV-negative 1 year following the insemination and subsequently had condomless sex with her HIV-infected partner (*29*).

Because of the increased efficacy of IVF compared with IUI or natural conception (*30*), IVF might afford less cumulative risk, since the number of exposures to the infected partner’s sperm is likely to be fewer. Risk for HIV transmission per IVF cycle is estimated to be similar to that with IUI (*4*). However, it is unknown whether there is a benefit to IVF compared with IUI in terms of HIV risk reduction that might offset the known surgical risk and financial cost associated with IVF if it is not being used for treatment of infertility. It is also not known whether intracytoplasmic sperm injection might further reduce the risk for transmission. As with IUI, transmission risk associated with IVF can be reduced with sperm washing, use of HAART by the infected male partner, and use of PrEP by the uninfected female partner during periods of potential exposure to HIV-infected sperm (daily while attempting conception, ideally beginning approximately 20 days before exposure) (*3*,*31*).

## Conclusion

There is considerable new information about prevention of HIV transmission in HIV-discordant couples since 1990 when CDC recommended against insemination with semen from HIV-infected men (*7*). Insemination with sperm from a donor who does not have HIV infection is the safest option for an HIV-uninfected woman with an HIV-infected male partner to conceive. However, current evidence suggests that the risk for transmission from an HIV-infected male partner to an HIV-uninfected female partner is low if appropriate risk-reduction strategies are implemented. As data regarding the safety and effectiveness of semen processing emerges, the risk profile for each treatment option will be further defined. HIV-discordant couples who desire to conceive might wish to discuss treatment options with a medical provider who can explain the risks and benefits of different treatment modalities as they apply to the couple’s specific situation before attempting conception.

**Summary**

**What is already known about this topic?**

For HIV-discordant couples (in which the man is HIV-infected and the woman is not HIV-infected) who wish to conceive a biological child, strategies to minimize the risk for sexual transmission are needed. In 1990, CDC recommended against insemination with semen from HIV-infected men.

**What is added by this report?**

Recent data regarding the safety of semen processing suggest that such processing is a viable option for HIV discordant couples attempting conception. The risk for transmission from an HIV-infected man to an HIV-negative woman is low if appropriate risk-reduction strategies, such as the use of highly active antiretroviral therapy, antiretroviral preexposure prophylaxis, and sperm washing are implemented. Recent evidence suggests that discordant couples might consider condomless intercourse timed to coincide with ovulation or intrauterine insemination of the woman or in vitro fertilization in combination with sperm washing after discussing the risks and benefits of each option with a medical provider.

**What are the implications for public health practice?**

As further data emerge, the risk profile for each treatment option will be further defined. HIV-discordant couples who desire to conceive might wish to discuss treatment options with a medical provider who can explain the risks and benefits of different treatment modalities as they apply to the couple’s specific situation before attempting conception.
