# Erratum: Vol. 66, No. 21

**DOI:** 10.15585/mmwr.mm6632a7

**Published:** 2017-08-18

In the report, “Strategies for Preventing HIV Infection Among HIV-Uninfected Women Attempting Conception with HIV-Infected Men — United States,” on page 555, the first full paragraph should have read as follows: “Condomless intercourse is associated with the highest risk for HIV transmission. The risk for male-to-female transmission in HIV-discordant couples has been estimated as approximately **8 per 10,000 episodes of condomless intercourse** (*10*). This estimation of risk is based, however, on natural history studies of couples before routine availability of HIV viral load measure­ments and HAART, and might vary widely with characteris­tics of the man and woman, including the presence of other sexually transmitted diseases, inflammation within the genital tract, and viral load of the infected partner (*10*). Some studies suggest a parallel reduction in plasma and semen viral loads (*11*), but other evidence suggests that plasma and semen viral loads might not correlate (*12*); men with undetectable plasma viral loads have had virus isolated from their semen (*13*). **Nonetheless, in a study that assessed sexual transmission risk during condomless intercourse in persons treated with HAART, the risk was lower among persons treated with HAART than among those not treated (hazard ratio 0.04, 95% confidence interval [CI] = 0.01–0.27) (*14*); not all treated were necessarily fully suppressed at the estimated time of transmission. Based on this study, for discordant couples in which the man is treated with HAART, the postulated risk for transmission to a female partner during condomless intercourse would be 0.32 per 10,000 exposures (95% CI = 0.06–1.7) (*10*).** In addition to viral suppression with HAART, the risk for sexual transmission can be further reduced by minimizing exposure frequency and limiting condomless intercourse to time of ovulation, thereby maximizing the chance of concep­tion, and by use of PrEP by the uninfected partner (*3*).”

